# Nonbacterial Microflora in Wastewater Treatment Plants: an Underappreciated Potential Source of Pathogens

**DOI:** 10.1128/spectrum.00481-23

**Published:** 2023-05-24

**Authors:** Sujani Ariyadasa, William Taylor, Louise Weaver, Erin McGill, Craig Billington, Isabelle Pattis

**Affiliations:** a Institute of Environmental Science and Research, Christchurch, New Zealand; Connecticut Agricultural Experiment Station

**Keywords:** wastewater treatment plant, nonbacterial microflora, shotgun metagenomic sequencing, virus, archaea, protozoa, fungi, environmental and human health, One Health

## Abstract

Wastewater treatment plants (WWTPs) receive and treat large volumes of domestic, industrial, and urban wastewater containing pathogenic and nonpathogenic microorganisms, chemical compounds, heavy metals, and other potentially hazardous substances. WWTPs play an essential role in preserving human, animal, and environmental health by removing many of these toxic and infectious agents, particularly biological hazards. Wastewater contains complex consortiums of bacterial, viral, archaeal, and eukaryotic species, and while bacteria in WWTP have been extensively studied, the temporal and spatial distribution of nonbacterial microflora (viruses, archaea, and eukaryotes) is less understood. In this study, we analyzed the viral, archaeal, and eukaryotic microflora in wastewater throughout a treatment plant (raw influent, effluent, oxidation pond water, and oxidation pond sediment) in Aotearoa (New Zealand) using Illumina shotgun metagenomic sequencing. Our results suggest a similar trend across many taxa, with an increase in relative abundance in oxidation pond samples compared to influent and effluent samples, except for archaea, which had the opposite trend. Additionally, some microbial families, such as *Podoviridae* bacteriophages and Apicomplexa alveolates, appeared largely unaffected by the treatment process, with their relative abundance remaining stable throughout. Several groups encompassing pathogenic species, such as *Leishmania*, *Plasmodium, Toxoplasma, Apicomplexa,* Cryptococcus*, Botrytis,* and *Ustilago*, were identified. If present, these potentially pathogenic species could be a threat to human and animal health and agricultural productivity; therefore, further investigation is warranted. These nonbacterial pathogens should be considered when assessing the potential for vector transmission, distribution of biosolids to land, and discharge of treated wastewater to waterways or land.

**IMPORTANCE** Nonbacterial microflora in wastewater remain understudied compared to their bacterial counterparts despite their importance in the wastewater treatment process. In this study, we report the temporal and spatial distributions of DNA viruses, archaea, protozoa, and fungi in raw wastewater influent, effluent, oxidation pond water, and oxidation pond sediments by using shotgun metagenomic sequencing. Our study indicated the presence of groups of nonbacterial taxa which encompass pathogenic species that may have potential to cause disease in humans, animals, and agricultural crops. We also observed higher alpha diversity in viruses, archaea, and fungi in effluent samples than in influent samples. This suggests that the resident microflora in the wastewater treatment plant may be making a greater contribution to the diversity of taxa observed in wastewater effluent than previously thought. This study provides important insights to better understand the potential human, animal, and environmental health impacts of discharged treated wastewater.

## INTRODUCTION

Wastewater treatment plants (WWTPs) are important sources and reservoirs for highly diverse microbial communities, including bacteria, viruses, archaea, and eukaryotes (1). These microbial communities not only affect the efficiency and stability of the WWTP but also may impact human health and the environment upon discharge of wastewater into the receiving environment. Therefore, studying wastewater microflora is of public health and ecological significance (2). Despite the key role of these microflora in WWTPs and their impacts on environmental and human health, variations in temporal and spatial distribution at different stages of the wastewater treatment process are poorly understood. In addition, recent advances in culture-independent molecular techniques and computational tools have enabled in-depth investigation of previously unknown or uncultivable WWTP microbial populations. However, the vast majority of these studies have focused only on bacteria (3), with little information available on the other microflora of WWTPs, such as viruses, archaea, and nonalgal eukaryotes.

Viruses are estimated to comprise a large proportion of the nonbacterial microflora in WWTPs, reaching 10^8^ to 10^10^ particles mL^−1^, which is 10- to 1,000-fold higher in concentration than natural aquatic habitats (4). Bacteriophages can directly impact bacterial and archaeal community composition through host cell lysis (5, 6) and may be responsible for about 40% of alterations in the anaerobic digester prokaryotic community (6). For example, a recent study reported that WWTP bacteriophages infected a wide array of bacterial and archaeal phyla, including functional organisms regulating nitrogen removal and carbon cycling (7). Bacteriophages are also known to act as vehicles for horizontal gene transfer and recombination in bacteria, resulting in the spread of virulence and antibiotic resistance genes in the environment (8). Studies have shown that a significant portion of the genomic sequences found in natural environments are of bacteriophage origin (9). Additionally, WWTPs harbor giant viruses such as *Mimiviridae* and *Phycodnaviridae* that can be pathogenic to a range of protists and algae (10).

Archaea are a significant element of the nonbacterial microflora in WWTPs whose diversity and community composition remain understudied. Emerging evidence suggests that WWTP archaeal communities are indispensable both functionally and ecologically due to their methanogenic and ammonia-oxidizing properties (11–13). Pan et al. (14) showed that ammonia-oxidizing archaea dominate over nitrogen-oxidizing bacteria during the winter season, indicating their importance in nitrogen transformation processes, particularly under unfavorable weather conditions. Similarly, many extremophilic archaea can thrive in a wide range of hyperthermal, saline, metallic, acidic, or alkaline environments that are inhospitable to other microorganisms; thus, they may be important in the bioremediation of heavily contaminated industrial wastewater inputs to the WWTP (14, 15). Methanogenic archaea play a vital role in the removal of organic compounds from heavily polluted wastewater with the associated production of gaseous methane that could be utilized as an energy alternative to fossil fuels (12).

Eukaryotes such as protozoa, fungi, parasites, and heterokonts (algae) also play vital roles in WWTP processes. As principal predators of bacteria, protozoa not only shape the bacteria community composition but also improve sludge sedimentation and effluent water quality (16, 17). Some protozoa are used as indicators of treatment efficacy; however, others can be pathogenic (16). Fungi such as Ascomycota can promote wastewater denitrification as well as cellulose degradation (17). Algae have been suggested as cost-effective, energy-efficient alternatives to activated sludge processing and could be utilized for biofuel production due to their ability to utilize inorganic carbon, nitrogen, and phosphorous in wastewater (18).

Based on direct environmental DNA extraction, shotgun metagenomic sequencing (MGS) has emerged as a powerful tool to interrogate the phylogenetic structure and function of complex microbial communities present in WWTPs (19, 20). Shotgun MGS can help mitigate some of the drawbacks of other approaches, including low cultivability of most WWTP microbes, low primer efficiencies and PCR bias of amplicon approaches, and the difficulty of taxonomic identification using microscopy (19, 21). MGS can help better understand the processes within WWTPs, providing information on the relationships and interactions between microflora and physicochemical factors (22, 23). Shotgun MGS studies have also led to the identification of uncultured WWTP microbes that are of environmental, biotechnological, and pharmaceutical importance (21, 22). However, due to cost limitations, many studies still rely on amplicon sequencing with limited taxonomic identification sensitivity.

In the present study, we analyzed wastewater samples obtained from a municipal New Zealand WWTP plant, using shotgun MGS to determine the composition of nonbacterial microflora at different stages of the treatment process, raw sewage influent (INF; postscreened), treatment plant effluent (EFF; post-primary settlement and secondary treatment via trickling filter beds and clarifiers), tertiary polishing ponds final effluent (POND), and sediments of the tertiary ponds (SED). We provide an overview of the nonbacterial WWTP microflora at various stages of the treatment process and discuss the temporal and spatial distribution shifts of nonbacterial microflora at each treatment stage as well as their potential ecological significance. These results will help understand the often-overlooked nonbacterial microflora of municipal wastewater and inform future risk assessments for vector transmission, biosolids reuse, and discharge into waterways.

## RESULTS

### Nonbacterial community composition.

**FIG 1:**
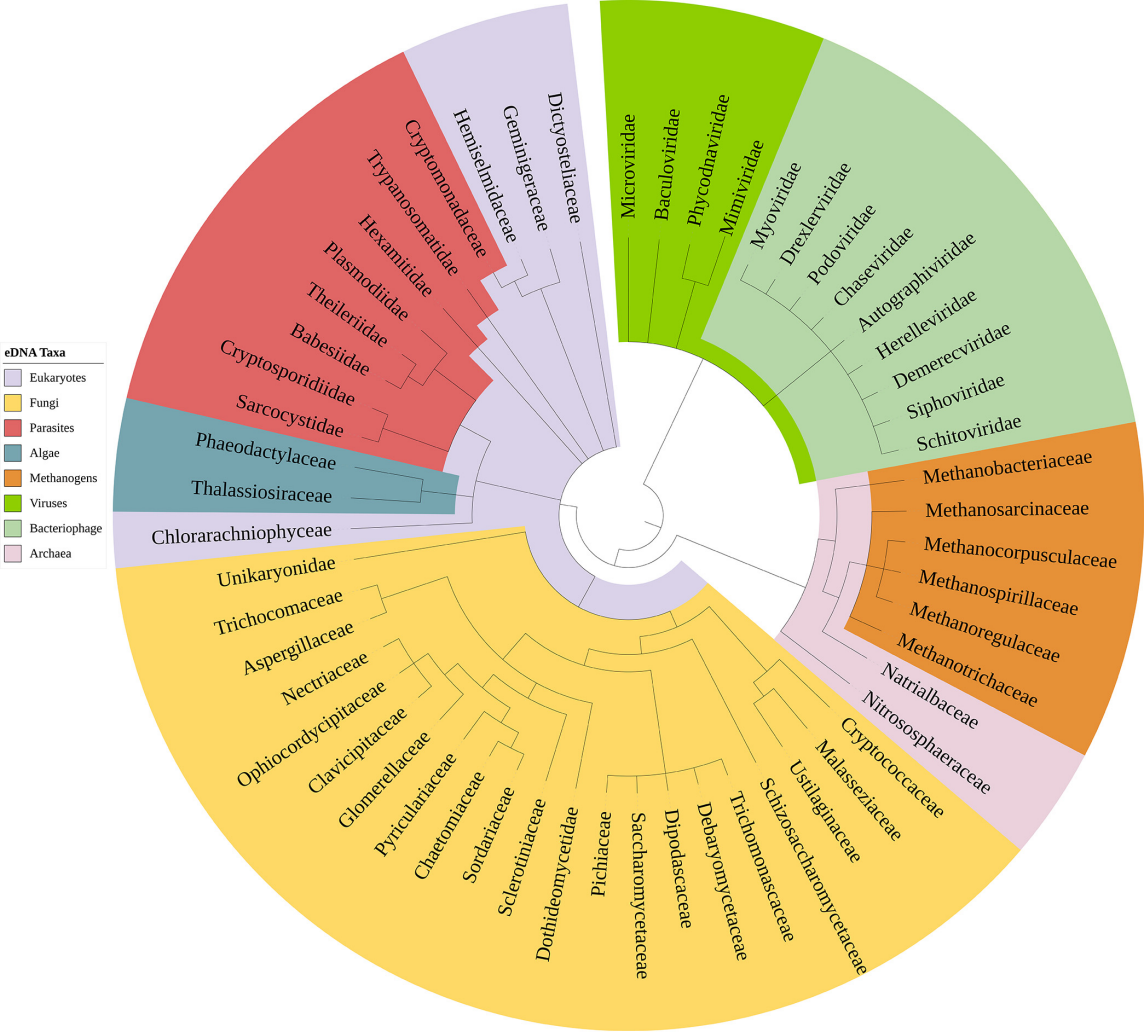
Tree of Life of identified nonbacterial families assigned across all samples.

Shotgun MGS data from wastewater samples collected from INF, EFF, POND, and SED of the WWTPs yielded an average total of 74.4 million reads per sample. Taxonomically classified reads were, as expected (see Table S1 in the supplemental material), comprised mainly of bacterial reads (98.8%), while the remainder were comprised of archaea (44.6%), viruses (31.7%), fungi (16.8%), and other eukaryotes, including protozoa and algae (7.4%). The composition of the nonbacterial taxa identified in the WWTP samples is shown in Fig. 1.

### Viruses.

**FIG 2:**
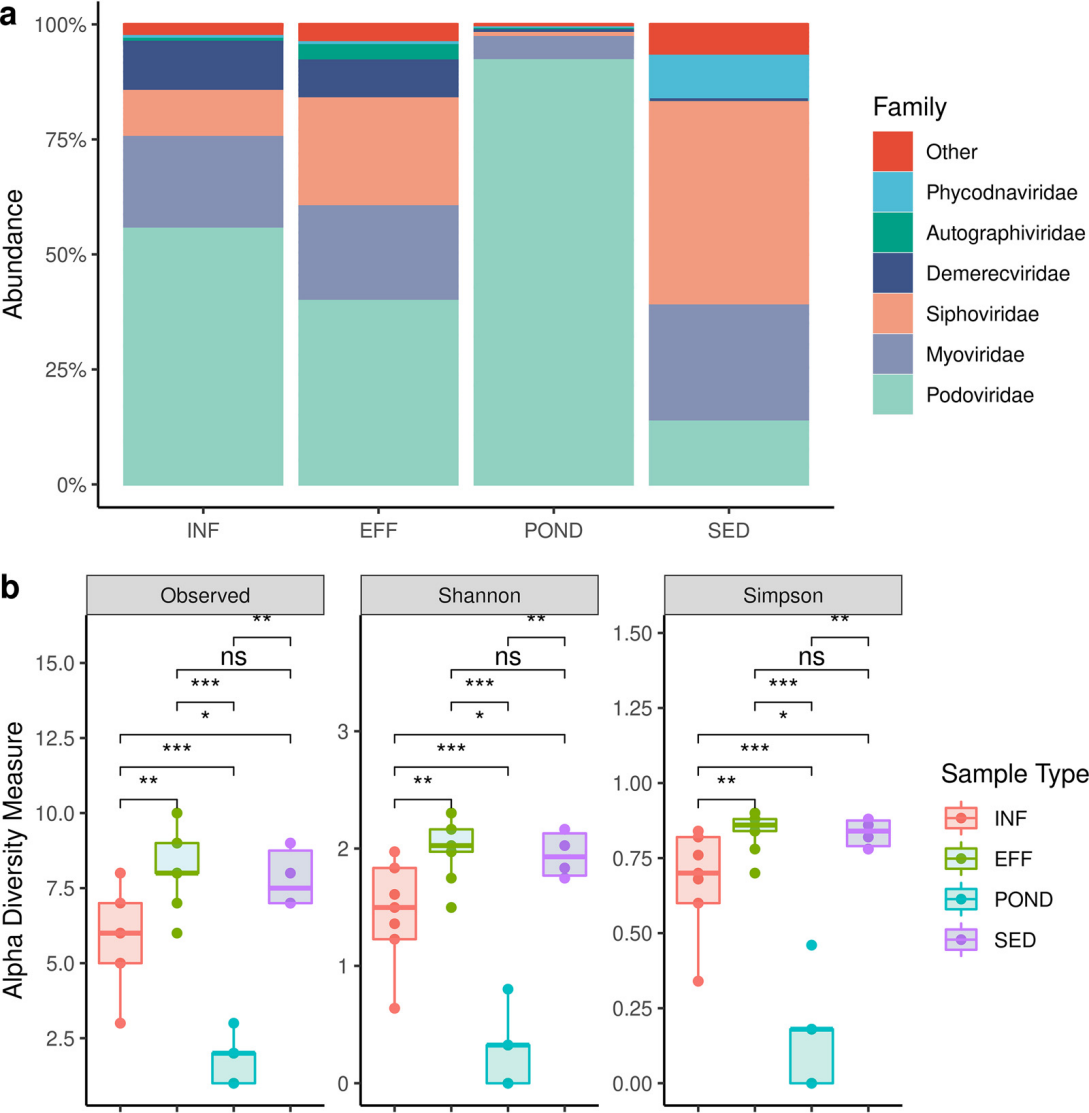
Wastewater virome. (a) Relative abundance of viral families. (b) Viral alpha diversity. Asterisks indicate Wilcoxon signed-rank test significance values. *, *P ≤ *0.05; **, *P ≤ *0.01; ***, *P ≤ *0.001; ****, *P ≤ *0.0001; ns, not significant.

The viral taxa were dominated by bacteriophage sequences with a minor nonbacteriophage viral component. Among bacteriophages detected, the families associated with enteric bacteria (23) were the most abundant, *Podoviridae*, *Myoviridae*, and *Siphoviridae* (Fig. 2a). A total of 23 *Podoviridae*, 78 *Myoviridae*, and 63 *Siphoviridae* bacteriophage sequences were identified. INF and EFF samples were dominated by crAssphage, while the relative abundance of *Myoviridae* and *Siphoviridae* decreased throughout the treatment process, and the relative abundance of *Podoviridae* was increased in the POND samples. *Myoviridae* and *Siphoviridae* were highly prevalent in SED samples; however, it should be noted that SED accumulates over a long period of time (several years), and no inference can be made on removal or increase relative to the other sample types (Fig. 2a and Table 1). SED samples instead provide a snapshot of historical inputs of organisms that can survive in mostly microaerobic and anaerobic environments.

**TABLE 1:** Percentage relative abundances of virus, archaea, protozoa/algae, and fungi at different stages of the wastewater treatment process^*a*^

Community component	Relative abundance (%) in:			
	INF	EFF	POND	SED
Viruses				
*Podoviridae*	56.1 (±13.1)	40.4 (±4.0)	92.7 (±4.0)	13.6 (±7.8)
*Myoviridae*	19.4 (±4.6)	20.0 (±2.7)	4.6 (±2.9)	25.2 (±11.5)
*Siphoviridae*	10.0 (±2.4)	24.0 (±2.2)	1.4 (±0.6)	44.8 (±13.4)
Archaea				
*Methanobacteriaceae*	56.4 (±4.0)	46.4 (±4.0)	22.7 (±3.1)	46.7 (±3.0)
*Methanosarcinaceae*	17.5 (±4.0)	33.2 (±5.0)	24.8 (±3.1)	13.0 (±3.0)
Protozoa				
Apicomplexa	57.2 (±7.2)	46.9 (±1.1)	51.5 (±5.8)	64.0 (±3.8)
*Plasmodium*	15.4 (±3.6)	19.7 (±0.7)	26.0 (±2.5)	35.3 (±4.0)
*Theileria*	1.9 (±1.1)	3.2 (±0.5)	2.1 (±0.7)	35.3 (±4.0)
*Cryptosporidium*	4.7 (±3.0)	3.1 (±0.7)	0.6 (±0.1)	3.8 (±1.7)
*Babesia*	10.1 (±8.5)	4.5 (±0.7)	0.9 (±0.2)	1.1 (±1.2)
*Toxoplasma*	24.0 (±6.5)	14.7 (±1.2)	17.4 (±6.5)	17.9 (±2.8)
Cercozoa	6.0 (±2.2)	9.1 (±1.3)	15.6 (±2.0)	13.2 (±3.6)
Euglenozoa	11.6 (±5.9)	6.1 (±1.3)	1.7 (±0.2)	1.7 (±1.1)
Fornicata	5.5 (±2.4)	0.1 (±0.1)	0.1 (±0.2)	0.1 (±0.2)
Microsporidia	6.9 (±2.4)	2.0 (±0.4)	2.8 (±0.6)	2.8 (±1.5)
Fungi				
Ascomycota	99.0 (±0.3)	95.4 (±1.0)	72.1 (±3.7)	73.2 (±5.0)
Basidiomycota	0.9 (±0.3)	4.6 (±1.0)	27.9 (±3.7)	26.8 (±5.0)
*Saccharomyces*	90.8 (±2.4)	27.4 (±12.2)	2.2 (±1.2)	1.0 (±0.8)
*Malassezia*	0.6 (±0.2)	0.7 (±0.2)	13.6 (±3.1)	2.8 (±1.4)
*Eremothecium*	0.7 (±0.1)	9.6 (±0.8)	2.8 (±0.4)	3.6 (±1.2)
Cryptococcus	0.3 (±0.1)	1.4 (±0.5)	7.2 (±0.5)	3.2 (±1.0)
*Ustilago*	0.05 (±0.03)	1.0 (±0.3)	1.0 (±0.3)	17.1 (±3.5)
*Botrytis*	0.1 (±0.1)	1.4 (±0.2)	4.3 (±0.5)	16.5 (±1.9)
Fusarium	0.2 (±0.1)	5.2 (±1.2)	6.1 (±0.4)	6.9 (±1.2)
*Drechmeria*	0.3 (±0.2)	29.2 (±11.4)	0.8 (±0.3)	4.5 (±1.1)
Aspergillus	0.7 (±0.1)	1.1 (±0.4)	2.4 (±0.5)	4.0 (±1.5)

a INF, influent; EFF, effluent; POND, pond water; SED, pond water sediment. Standard deviations are indicated within the parentheses.

*Autographiviridae*, *Demerecviridae*, *Chaseviridae*, *Herelleviridae*, and *Adenoviridae* were also identified in INF samples, with reduced relative abundances (<0.01%) after treatment, possibly indicating the removal of their hosts. The highest levels of *Phycodnaviridae* and *Mimiviridae* were detected in POND samples. There was a higher viral diversity in EFF samples than in INF and SED samples, whereas POND had the least diversity (alpha diversity measure, EFF > SED > INF > POND) (Fig. 2b). Except for between EFF and SED samples, the viral alpha diversity was significantly different between all sample types (*P < *0.05).

### Archaea.

**FIG 3:**
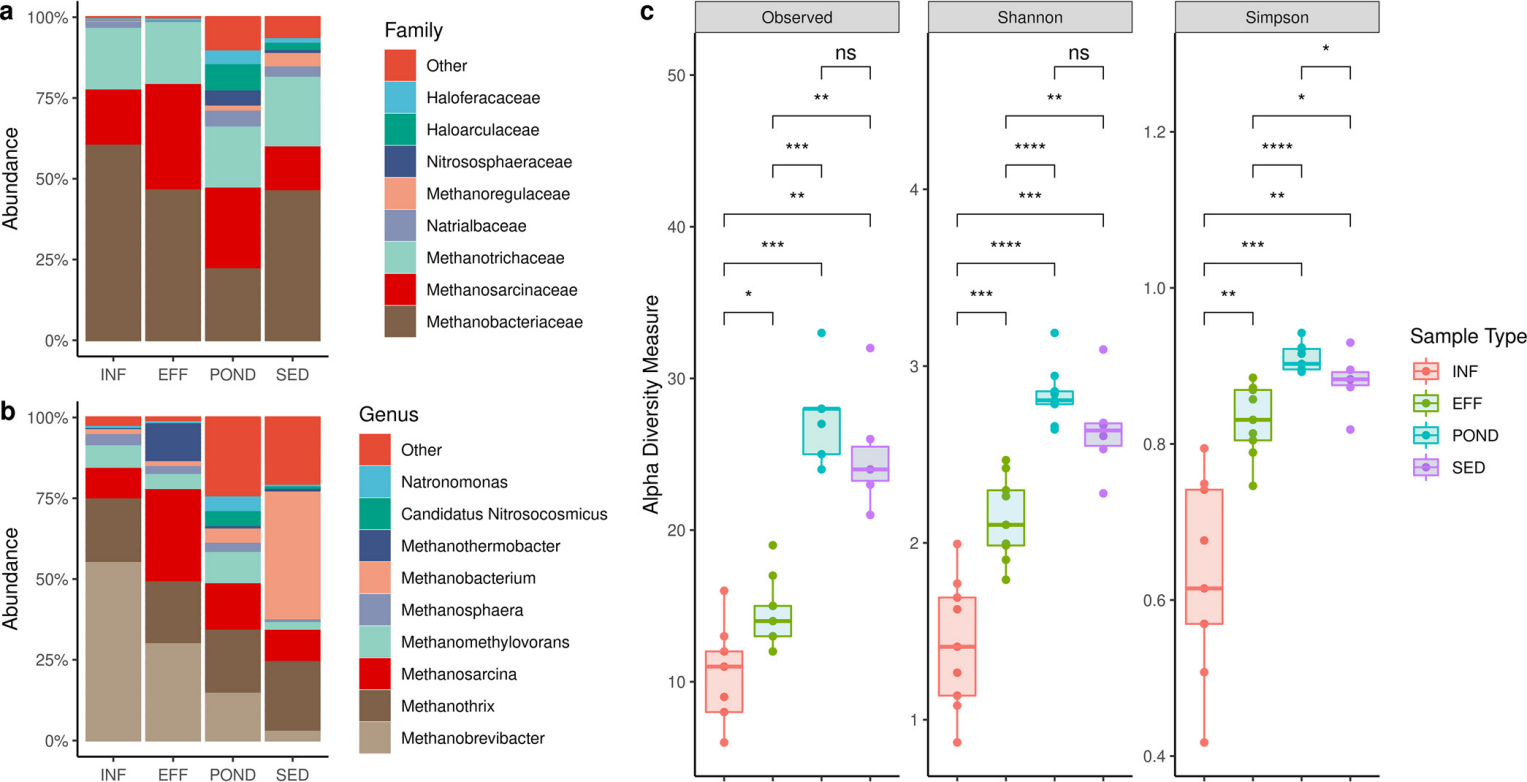
Wastewater archaea. (a) Relative abundance of archaeal families. (b) Relative abundance of archaeal genera. (c) Archaeal alpha diversity. Asterisks indicate Wilcoxon signed-rank test significance values. *, *P ≤ *0.05; **, *P ≤ *0.01; ***, *P ≤ *0.001; ****, *P ≤ *0.0001; ns, not significant.

The archaeal communities in INF, EFF, POND, and SED samples were dominated by three methanogenic families, *Methanobacteriaceae*, *Methanosarcinaceae*, and *Methanotrichaceae* (Fig. 3). *Methanobacteriaceae* constituted 56.4 ± 4.0% of the archaeal community of INF samples and decreased in relative abundance over the course of the wastewater treatment and then proportionately increased in SED samples (Table 1).

Despite this, *Methanobacteriaceae* remained the most abundant archaeal family across all treatment stages and were represented by the genera *Methanobrevibacter*, *Methanothermobacter*, *Methanosphaera*, and *Methanobacterium* (Fig. 3a). Conversely, sequences from *Methanosarcinaceae*, which consist of the genera *Methanosarcina* and *Methanomethylovorans*, increased in relative abundance from INF to EFF and remained largely unchanged in POND samples before decreasing in the SED samples (Table 1).

The relative abundance of *Methanotrichaceae* remained largely unchanged, and *Methanothrix* (Fig. 3b) was the most prevalent *Methanotrichaceae* genus detected. The highest archaeal alpha diversity was reported in POND and SED samples, as indicated by an increase in alpha diversity measurements (Fig. 3c). Except between the POND and SED samples, the archaeal alpha diversity was statistically significantly different between all sample types (*P *< 0.05).

### Protozoa.

**FIG 4:**
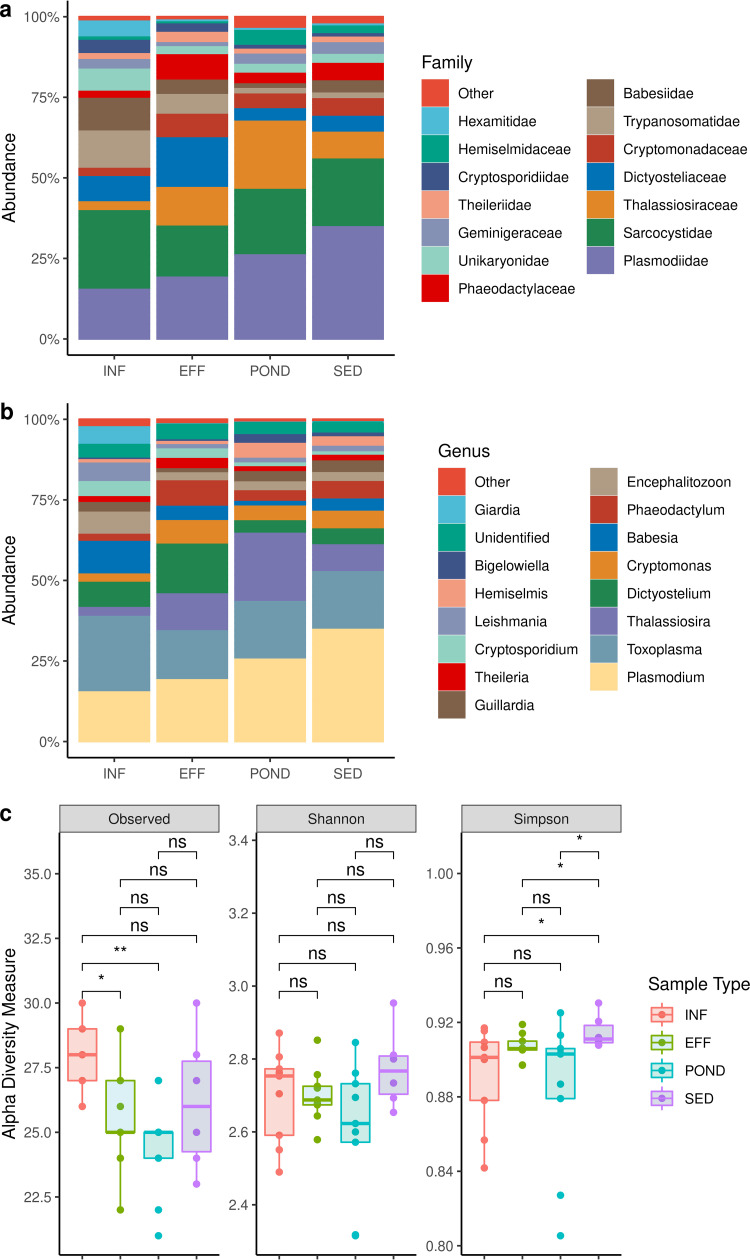
Wastewater protozoa. (a) Relative abundance of protozoan families. (b) Relative abundance of protozoan genera. (c) Protozoan alpha diversity. Asterisks indicate Wilcoxon signed-rank test significance values. *, *P ≤ *0.05; **, *P ≤ *0.01; ***, *P ≤ *0.001; ****, *P ≤ *0.0001; ns, not significant.

In addition to viruses and archaea, WWTP samples included a diverse population of protozoa (Fig. 4). Apicomplexa were the most dominant in the city’s wastewater, followed by Cercozoa, Evosea, Euglenozoa, Microsporidia, and Fornicata. Across all samples, Apicomplexa comprised at least half of the total protozoan population; 57.2 ± 7.2% in INF, 46.9 ± 1.1% in EFF, 51.5 ± 5.8% POND, and 64.0 ± 3.8% SED. At the genus level, Apicomplexa was mainly represented by *Plasmodium* (P. vivax, P. cynomolgi, P. knowlesi, P. relictum, and P. yoelii), *Toxoplasma* (T. gondii), *Cryptosporidium* (C. parvum), Giardia (G. lamblia), *Babesia* (B. bovis, B. bigemina) and *Theileria* (T. annulata, T. parva). Among these, the relative abundance of *Plasmodium* sequences (Table 1) was higher in POND and SED samples, while *Theileria* sequences were more prevalent in SED samples. The relative abundances of *Cryptosporidium* and *Babesia* were reduced in EFF and POND samples and increased in SED compared to POND samples. Overall, *Toxoplasma* relative abundance decreased from INF to POND, despite an observed rise from EFF to POND (Fig. 4b and Table 1).

In contrast to Apicomplexa, the abundance of Cercozoa, a phylum consisting of amoeboid and flagellated protists, increased (24) from INF to EFF and POND samples (Table 1); however, reduced in abundance but persisted in SED samples (Table 1). The abundance of Euglenozoa, a phylum comprising the human parasitic genera *Trypanosoma* and *Leishmania* (25), decreased throughout the treatment process (Table 1). No change in the prevalence of Euglenozoa was observed between POND and SED. In contrast, intracellular parasites Fornicata and Microsporidia relative abundance decreased more rapidly between INF and EFF (Table 1). The highest alpha diversity for protozoa was observed in INF samples, while Shannon and Simpson diversity indices were highest in SED samples; however, their statistical significance across indices was not consistent (Fig. 4c).

### Fungi.

**FIG 5:**
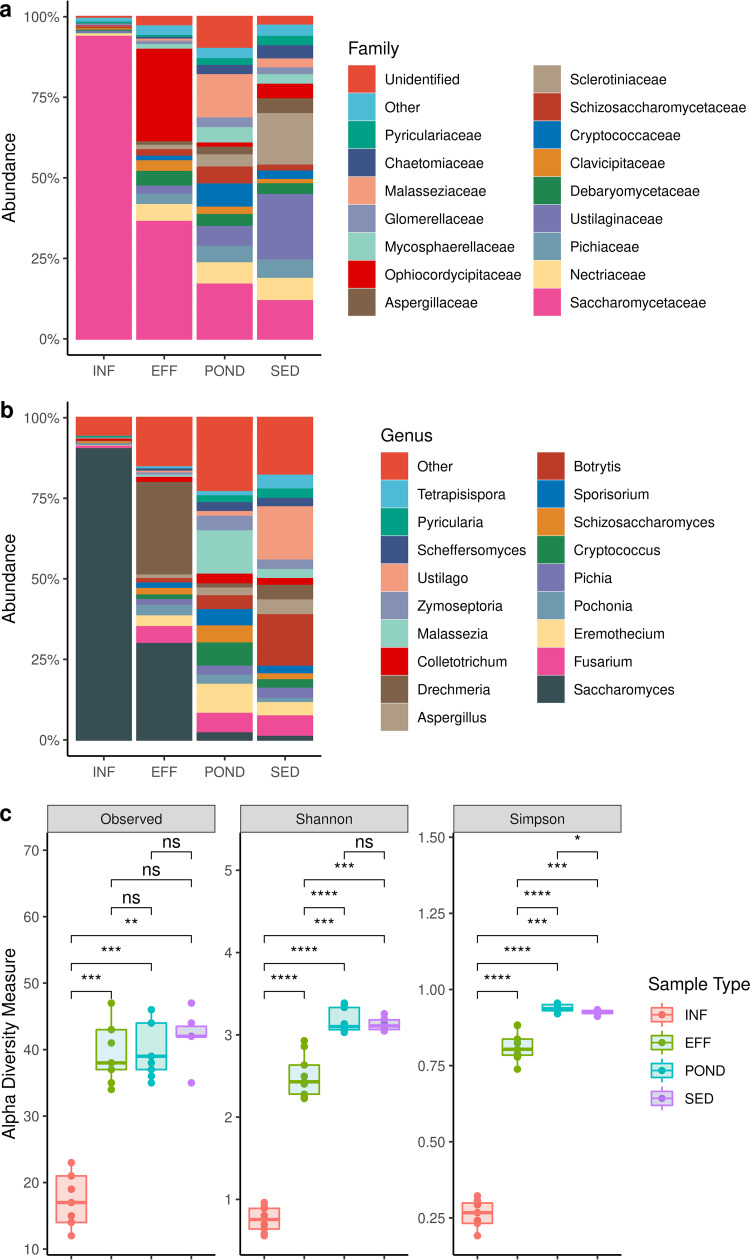
Wastewater fungi. (a) Relative abundance of fungal families. (b) Relative abundance of fungal genera. (c) Fungal alpha diversity. Asterisks indicate Wilcoxon signed-rank test significance values. *, *P ≤ *0.05; **, *P ≤ *0.01; ***, *P ≤ *0.001; ****, *P ≤ *0.0001; ns, not significant.

The fungal community, or mycobiome, of wastewater consisted of the phyla Ascomycota and Basidiomycota. Ascomycota comprised over 90% of the fungi in INF and EFF samples and were comparatively less prevalent in POND and SED samples (Table 1). In contrast, the relative abundance of Basidiomycota was higher in POND and SED samples (Table 1). Ascomycota consisted mainly of the families *Saccharomycetaceae*, *Ophiocordycipitaceae*, *Sclerotiniaceae*, Aspergillaceae, and *Nectriaceae*, whereas Basidiomycota were mainly represented by *Ustilaginaceae* and *Malasseziaceae* (Fig. 5a).

At the genus level, INF samples were dominated by *Saccharomyces* (S. cerevisiae, S. eubayanus, S. paradoxus) (Fig. 5b and Table 1); however, its prevalence sharply decreased in subsequent treatment stages. *Drechmeria*, a nematophagous *Ascomycetes* fungal genus (26), showed increased abundance in EFF samples compared to INF samples and decreased again in POND and SED samples (Table 1). POND samples were dominated by the Basidiomycota genus *Malassezia* (M. restricta, an opportunistic fungal pathogen of the human skin microbiota) (27), plant-pathogenic genus *Eremothecium* (E. gossypii, E. sinecaudum, E. cymbalariae), human-pathogenic Cryptococcus (C. neoformans, C. gattii), and the human and plant-pathogenic genus Fusarium (F. oxysporum, F. graminearum, F. venenatum). SED samples mainly consisted of plant-pathogenic fungal genera such as *Ustilago* (U. maydis) and *Botrytis* (B. cinerea), as well as Fusarium (F. oxysporum, F. venenatum), *Drechmeria* (D. coniospora), and Aspergillus (A. fumigatus) (Fig. 5b; Table 1). The alpha diversity for fungal taxa was lowest in INF samples due to the high abundance of *Saccharomyces* (Fig. 5c), while the highest alpha diversity was present in POND samples. Statistically significant alpha diversity differences (*P* < 0.05) were seen between each treatment stage, except in EFF and POND, POND and SED, and EFF and SED (observed) and POND and SED (Shannon index).

## DISCUSSION

WWTPs are biotechnological systems that have a crucial role in preserving human, animal, and environmental health. WWTPs receive and treat large volumes of domestic, industrial, and urban wastewater (~60% of global wastewater annually [28]). Discharge of treated effluent occurs predominantly in waterways, with a smaller proportion applied to land. Analyzing municipal wastewater at the WWTPs has become indispensable for monitoring disease, with a prime example being the monitoring of COVID-19 community prevalence and the emergence of SARS-CoV-2 variant mutations during the pandemic (29). The effectiveness of WWTPs depends on the physical and biological processes performed by both bacterial and nonbacterial microbial flora present in these systems (30). However, many studies only focus on the bacterial microflora of WWTPs, leading to an important knowledge gap in how these systems operate.

### The wastewater virome is dominated by bacteriophages of gut origin.

The most abundant bacteriophages detected in the wastewater samples were well-established inhabitants of the human gut, *Podoviridae*, *Myoviridae*, and *Siphoviridae* (31). These are morphologically diverse tailed somatic coliphages belonging to the *Caudovirales* that have double-stranded DNA (dsDNA) genomes of ~10 to 100 kb (32). *Podoviridae* bacteriophages have short tails and isometric capsids of ~65 nm, whereas *Myoviridae* bacteriophages have long contractile tails and capsids up to 100 nm in length. Members of *Siphoviridae* have long noncontractile tails with isometric capsids of ~60 nm (33). A high prevalence of *Myoviridae* and *Podoviridae* families have been observed in municipal wastewater samples collected from industrialized countries such as Japan and Saudi Arabia (4, 34). In addition to the conventional somatic coliphages (*Podoviridae*, *Myoviridae*, and *Siphoviridae*), newly characterized somatic coliphage families, such as *Demerecviridae*, *Drexlerviridae*, *Autographiviridae*, and *Ackermannviridae*, were detected in high abundance in INF samples (33), consistent with their high fecal input.

Influent samples were dominated by crAssphage, the most abundant bacteriophage in the human gut. Although crAssphage are very similar to the members of the *Podoviridae* family morphologically, recent sequence- and protein-based studies have identified crAssphage as a novel family within the order *Caudovirales* (35). crAssphage-like bacteriophages have been identified in human gut samples from various geographical locations. Although crAssphage are more frequently associated with the gut microbiomes of industrialized countries (36), they have been identified globally in sewage samples except in the Antarctic region (31). Studies have shown that the crAssphage comprise up to 1.68% of total human fecal metagenomic reads and likely infect commensal gut bacteria belonging to *Bacteroides* spp. (37). Despite this, a large proportion of viral metagenomic sequences are not identified due to the scarcity of bacteriophage genomic data in reference database systems (31). Additionally, RNA viruses, which could comprise an unknown proportion of bacteriophages, were not included in this study (38) The relative abundance of crAssphage decreased in POND samples, possibly due to the inactivation of most of their enteric bacterial hosts as indicated by our bacterial metagenomics study of the same samples (39). However, interestingly, crAssphage were still predominantly detected in EFF samples, indicating they was not eliminated by the initial treatment process. A recent study reported that crAssphage were detected across all WWTP treatment stages and showed the highest relative abundance in the presence of other fecal indicator organisms, Escherichia coli, enterococci, somatic coliphages, adenovirus, polyomavirus, and the HF183/BacR287 molecular indicator region (40). It is also known that the persistence of crAssphage in wastewater is independent of seasonal fluctuations (41). These results reinforce the suitability of crAssphage as a tool for surveilling human fecal contamination and the potential presence of other pathogens of human fecal origin in environmental water systems.

Bacteriophages associated with agriculturally significant hosts, such as *Geobacillus*, *Lactococcus*, and *Lactobacillus* spp., were also detected in INF samples, although in lower abundance than bacteriophages of human gut origin. *Geobacillus* are soil-dwelling, thermotolerant, spore-forming rod bacteria commonly isolated in a wide variety of environments such as stable manure, dairy waste, compost, and greenhouse soils (42). While *Geobacillus* bacteriophages can be detrimental in medical and industrial biotechnology applications due to their rapid infection rate, recent studies have shown that the enzymes encoded by these bacteriophages can be effectively utilized in removing biofilms from industrial fixtures and medical equipment (43). Additionally, the presence of bacteriophages infecting *Lactococcus* and *Lactobacillus* spp. could indicate dairy and food industry effluent entering the WWTPs or may be a result of human or animal dietary probiotic supplements. Another interesting finding was the presence of bacteriophages infecting potential plant pathogens (*Burkholderia*, *Dickeya*, *Erwinia*, *Xanthomonas*, and *Xylella*) in INF samples (44). Members of these bacterial genera can affect a wide range of crops and have resulted in significant economic damage due to crop loss (45). Previous biocontrol trials conducted using phytopathogenic bacteriophages have indicated that they can be used as a sustainable alternative to chemicals or antibiotics in controlling bacterial plant diseases (44). For example, the prophylactic application of *Xylella* bacteriophage cocktail Sano, Salvo, Prado, and Paz has been applied to grapevines to successfully treat Pierce’s disease (46). The *Xylella* bacteriophages Sano, Salvo, and Prado were detected in INF samples, suggesting that wastewater may be a useful source of bacteriophages with potential for biocontrol of plant pathogens.

Bacteriophages have an important role in the evolution of prokaryotes by facilitating horizontal gene transfer between bacteria, including those belonging to intestinal taxa such as *Proteobacteria*, *Bacteroidota*, *Actinomycetales*, and *Firmicutes*. Bacteriophages also contribute to the transmission of antimicrobial resistance (AMR) genes between bacteria and have been detected carrying AMR genes in both pristine and anthropogenic-impacted environments such as wastewater, river water, reclaimed water, and manure-amended soil (47). Interestingly, a study evaluating the bacterial and bacteriophage fractions found that AMR genes were generally more persistent in the bacteriophage fraction than in the bacterial fraction (39).

### Effluent samples consisted of viruses with enteric and environmental hosts.

The virome of EFF samples consisted of bacteriophages with enteric and environmental hosts. As discussed in the previous section, crAssphage dominate the EFF samples, followed by Mycobacterium bacteriophages. Mycobacteria are often isolated in a variety of aquatic habitats, including sewage sludge and known biofilm colonizers at air-water and air-soil interphases (48). Similar to our study, a high prevalence of Mycobacterium bacteriophages was observed in nonpotable reclaimed water samples in Florida, United States (49), and EFF samples collected from WWTPs in Japan (4), indicating that some viral communities in different WWTPs may be conserved. Bacteriophages infecting Pseudomonas, *Flavobacterium*, *Aeromonas*, Klebsiella, and *Lactococcus* spp. were also detected in EFF samples, consistent with a high abundance of environmental bacteria. In addition, EFF samples recorded the highest relative abundance of *Gordonia* bacteriophages compared to INF, POND, and SED samples. Their hosts, *Gordonia* spp., are found in many environments, including water and soil, and can cause skin infections in humans. *Gordonia* spp. have been isolated from activated sludge and are associated with foaming-related problems in WWTP, decreasing their effectiveness. Therefore, isolation and application of specific *Gordonia* bacteriophages to activated sludge systems could potentially be used to help ameliorate foaming-related issues in WWTPs (50). On the other hand, *Gordonia* spp. have been shown to degrade xenobiotics and other environmental pollutants in wastewater and may have the potential to be used for targeted treatment mitigations (51).

### Increased abundance of viruses with environmental hosts in POND and SED samples.

Nonbacteriophage viruses were more prominent in POND samples despite the significantly lower viral diversity than in other treatment stages. For example, two virus families belonging to nucleocytoplasmic large DNA viruses (NLDV), *Mimiviridae* and *Phycodnaviridae*, which infect algae, amoeba, and other protists, were detected in high abundance in POND samples. This may correspond to the increase of free-living amoeba and algal phyla such as Evosea, Euglenozoa, and Cercozoa under favorable environmental conditions in the ponds during spring and early summer. Warmer temperatures assist the excystation of otherwise dormant amoeba, increasing the number of actively feeding trophozoites in the environment (52). We presume that this contributed to a rise in *Mimiviridae* predation and intracellular replication, resulting in their increased abundance in POND samples (53). Our study also detected an increased relative abundance of *Vibrio* bacteriophages (a member of the *Podoviridae* family) in POND samples compared to INF. Environmental *Vibrio* spp. are typically isolated from saltwater/brackish water (54), and so, it is possible that aerosolized transmission of *Vibrio* spp. from the nearby estuary to the POND contributed to the observed increase.

### The archaeal metagenome is dominated by methanogens.

Methanogenic archaea are a diverse group of Euryarchaeota that play an important role in the global carbon cycle. Their energy metabolism is limited to methane produced from carbon dioxide and hydrogen gas, methanol, formate, acetate, and/or methylamines (55). It is known that methanogenic archaea produce ~1 billion tons of methane per year globally. According to previous studies, high abundances of methanogenic archaea have been associated with WWTPs receiving high volumes of industrial wastewater (55, 56). For example, the aceticlastic methanogenic family *Methanosarcinaceae* have been observed in dairy waste containing elevated levels of ammonia and volatile fatty acids and in brewery wastewater with high concentrations of acetate (56). Similarly, industrial wastewater containing high levels of volatile fatty acids is known to contain *Methanotrichaceae*, an aceticlastic methanogenic family of archaea (57). *Methanobacteriaceae*, a family of rumen methanogens found in cattle and sheep, is commonly associated with livestock effluent (56). In addition, recent studies have shown that the human gut archaeome predominantly consisted of *Methanobacteriaceae* (57, 58). As such, domestic sewage may also contribute to the methanogenic archaea present in wastewater. Understanding the methanogenic archaeal community composition and their variation also provides insight into the use of wastewater as an effective biomass for biomethane production. Conversion of biowaste into biomethane not only provides a cost-effective, environmentally friendly means of energy production but also significantly reduces undesirable methane leakage into the atmosphere (55). It is noteworthy that the WWTP discussed in this paper utilizes biogas produced onsite to power some of its vehicles.

### Parasitic protozoa persist throughout the treatment process.

Apicomplexa, a phylum consisting of diverse, obligatory intercellular parasites, represented nearly 60% of the protozoan microflora in raw wastewater (INF) received at the WWTP. Apicomplexa includes important human and vertebrate parasites of public health concern such as *Cryptosporidium*, Giardia, *Plasmodium*, *Toxoplasma*, and *Babesia.* Human gastroenteric illness and diarrhea caused by *Cryptosporidium* and Giardia are among the most prevalent waterborne parasitic protozoa-related outbreaks, with 48% of reported global waterborne protozoa outbreaks reported between 2011 and 2016 occurring in New Zealand, suggesting a high prevalence of disease in the country (59). Individuals infected with *Cryptosporidium* shed large numbers of oocysts in feces (up to 10^9^ oocysts per stool) that persist in cold, moist environments for up to 6 months (60, 61), and for individuals with giardiasis, up to 10^10^ cysts are shed per stool per day (60). The high relative abundances of gastrointestinal pathogenic protozoa in INF samples suggest community occurrence of cryptosporidiosis and giardiasis during the sampling period, which is in agreement with the seasonal peak of cryptosporidiosis notifications in New Zealand during spring, coinciding with the calving and lambing season (62, 63). This pattern may also reflect WWTP inputs from meat processing of calves and lambs. It should be noted that identification of *Cryptosporidium* and Giardia species was not at a low-enough taxonomic level to identify clinically relevant strains.

Our sequence data indicate that a large fraction of protozoan pathogens were removed throughout the wastewater treatment process (87% reduction in the relative abundance of *Cryptosporidium* from INF to POND samples). However, the presence of *Cryptosporidium* sequences in SED samples indicates potential for long-term deposition of oocysts and persistence in pond sediments. Low-level persistence of *Cryptosporidium* has previously been detected in sediment samples collected from an urban river in Christchurch, following cessation of sewage discharge during a large earthquake event from 2010 to 2011 (64). As such, the investigation of *Cryptosporidium* oocyst viability in sediment would be recommended prior to the potential application of this sediment to the environment. A recent study found viable *Cryptosporidium* oocysts in secondary (30%) and final effluent (40%) samples, highlighting the potential for *Cryptosporidium* transmission from treated wastewater samples (65). In contrast, the relative abundance of Giardia was reduced by >99% posttreatment, suggesting their effective removal, or sequestration, during the wastewater treatment process.

*Plasmodium* was another dominant genus of Apicomplexa detected in INF samples. plasmodia are unicellular eukaryotic insect-borne parasites that infect reptile, bird, and mammalian hosts and are known to cause mosquito-borne illnesses such as malaria (66). While the presence of plasmodia, such as P. malariae, P. vivax, and P. falciparum, was likely *Plasmodium* oocysts shed by infected hosts, as there is no local mosquito-borne transmission of these parasites among humans due to the absence of the *Anopheles* spp. of mosquito in New Zealand (67). However, the transmission of avian malaria (P. relictum) has been reported in 35 native and nonnative bird species (68, 69). There is a high prevalence of P. relictum in blackbirds, a common garden bird in urban areas, which is a likely main source of transmission to native birds due to shared habitats (70) and may be a potential source of *Plasmodium* identified in this study. In addition, the increase in *Plasmodium* relative abundance reported in EFF, POND, and SED suggests contamination of sedimentation tanks and polishing ponds with bird droppings. Therefore, these results provide important insight into the ecology and epidemiology of both avian and human malaria in New Zealand.

Another apicomplexan parasite of clinical significance identified in INF samples was *Toxoplasma*, the causative agent of toxoplasmosis in humans and other warm-blooded animals (71). The disease is transmitted in humans through the consumption of raw meat from infected animals, ingestion of contaminated water, or contact with soil-containing oocysts (71). According to our results, the relative abundance of *Toxoplasma* was largely unaffected by the treatment process, indicating its prevalence in treated wastewater as well as in sediments. These findings indicate that waterborne toxoplasma transmission may be a concern for both humans and animals in the region, particularly for New Zealand native wildlife. Studies show that toxoplasmosis has been detected in a variety of New Zealand endangered native species, including Hector’s and Māui dolphins, kiwi, kākā, and near-threatened red-crowned kakariki (72). It is possible that the prevalence of *Toxoplasma* in humans is underestimated due to the often-asymptomatic nature of the infection (71).

### The fungal metagenome reflected industrial and agricultural species.

The fungal metagenome of INF samples was dominated by *Saccharomyces*, a genus of fungi found in soil (73) and extensively used in the food and beverage industry, particularly in brewing and winemaking. Brewery and winemaking are two of the most popular industries in New Zealand, and reports show that the country has more breweries per capita (4.56 per 100,000 people) than other countries with well-established brewing industries such as Australia, the United Kingdom, and the United States (74). However, the relative abundance of *Saccharomyces* reduced significantly during the wastewater treatment process, indicating its effective removal. Other fungal genera increased in EFF samples, such as *Drechmeria*, *Botrytis*, Aspergillus, *Ustilago*, Cryptococcus, and *Malassezia*. Among these, *Drechmeria* is known for its unique predatory mechanism, which involves “nematode trapping” using mechanical or adhesive functions, parasitism of females and eggs, and endoparasitism of juvenile and adult nematodes (26). *Drechmeria* persisted in the wastewater treatment process despite a reduction in relative abundance and was also present in SED, indicating its deposition in the bottom of the polishing ponds, likely due to the high abundance of nematodes.

The relative abundance of *Botrytis*, Aspergillus, and *Ustilago* genera continued to increase from EFF to POND, possibly due to storm runoff containing contaminated soil entering the ponds. *Botrytis* and *Ustilago* were the most abundant fungal genera in SED samples, suggesting that their spores may accumulate in the sediments of the oxidation pond. *Botrytis* (B. cinerea) is a well-established pathogen of a wide range of horticultural crops such as grapes, kiwifruit, boysenberry, strawberry, and sweet cherry, all of which are significant for New Zealand’s trade economy (75). In 2010, the financial impact of botrytis bunch rot was estimated to be NZ $6,500/ha in wetter regions of New Zealand due to crop loss and control costs (76). Similarly, a common smut disease caused by *Ustilago* was recently identified in a cornfield in New Zealand (77). As such, the WWTP may act as a sink of plant-pathogenic fungi and a possible transmission route to the environment. However, further studies are required to confirm the viability and virulence of these fungi in wastewater and sediment samples.

### Limitations of the study.

This study does not represent the complete metagenome of the WWTP, particularly for viruses, as RNA was not sequenced. Furthermore, low percentages (INF, ~43%; EFF, ~16%; POND, ~14%; SED, ~4%) of the total sequence reads were assigned to known taxa, which can be attributed to the presence of previously unclassified novel organisms in the studied WWTP environments, DNA degradation in the environment, or taxa without complete RefSeq genomes available. Additionally, due to the nontargeted experimental design, low numbers of nonbacterial reads were classified, and as such, an additional targeted approach could increase the robustness and significance of these findings. Although our results indicate the presence of genera which include potential animal and plant pathogens in wastewater, more targeted investigations, including viability assays, are required to perform a comprehensive risk assessment on the impact these potential pathogens pose to human, animal, and environmental health. Also, additional factors such as the species, infectious dose, transmission mode, and virulence of the organism need to be considered to determine the potential disease burden of the pathogens identified in this study. Future investigations should include WWTP sludge and biosolids, both of which might accumulate potential pathogens.

### Conclusions.

This study shows that municipal WWTPs harbor complex communities of viruses, archaea, protozoa, and fungi and that the temporal and spatial distribution of these microflora varied throughout the wastewater treatment process. It is likely that the taxa present at different stages of the wastewater treatment process reflect abiotic factors characteristic of each treatment stage. An increase in the diversity of most taxa was found in oxidation pond water, indicating that wastewater discharge may contain many viral, archaeal, protozoan, and fungal taxa, both previously identified and novel. This was likely influenced by the residence time, leaching of sludge-accumulated organisms, and environmental factors. This also indicated that municipal wastewater may be a useful source of bacteriophages for use as biocontrol agents for crop and livestock diseases.

The raw wastewater received at the WWTP mainly consisted of domestic and industrial wastewater inputs containing human and animal fecal pollutants. Domestic and industrial sewage input to this WWTP is predicted to increase over the coming years with the city’s growing population. As such, these results indicate the need for improved effluent monitoring and new management strategies, such as on-site waste processing, value addition of industry and dairy waste, and biofuel generation. It also highlights the significance of implementing adequate regulatory policies to help mitigate the risk of overburdening WWTP capacity, which may lead to adverse effects on human, animal, and environmental health.

This study also provided insight into the nonbacterial pathogen removal efficiencies of the WWTPs. Our results showed that some potential pathogens were effectively removed following treatment, while others were unaffected. For example, the WWTPs optimally removed human adenovirus and Ascomycota and *Saccharomyces* spp., whereas poor removal efficiencies were observed for the parasitic protozoa *Toxoplasma*, *Plasmodium*, and other *Apicomplexa.* As such, the final treated effluent may have contained pathogenic taxa with the potential to cause disease in humans, animals, and agricultural crops. The threat that the presence of these potentially pathogenic taxa may pose to human and environmental health is currently unclear, and so, further work is warranted to understand this finding, particularly regarding land applications of biosolids and the discharge of treated wastewater into waterways. Further investigations into the removal efficiencies of parasitic protozoa may also be beneficial to further improve discharge water quality.

The monitoring of community composition dynamics of nonbacterial microflora may therefore be an important new tool to assist in managing and optimizing the removal of contaminants from wastewater and to inform future risk assessments for improving human, animal, and environmental health.

## MATERIALS AND METHODS

### Sample collection.

Sampling took place in spring and early summer of 2019 (November and December) in New Zealand as described previously (39). A wastewater treatment plant servicing ~134,000 households had 24-h composite samples taken from influent (INF) postscreens, treated effluent (EFF), tertiary oxidation pond effluent (POND), and sediments (SED) on three consecutive days. The treatment process includes an initial stage of removing nonorganic solids (by filtration and gravity), followed by removing remaining organic solids by sedimentation, converting nonsolid nutrients into microbial biomass, and subsequent separation. The plant removes carbonaceous nutrients only and does not treat for ammonia or other nutrients. Wastewater was collected as hourly composites (sampled every 15 min) using an automatic sampling device (Isco, Teledyne Technologies Inc., USA). After 24 h, the hourly composites were combined into 24-h samples, of which three replicate samples were taken. POND sediment (10 g) was collected as triplicate grab samples concomitant with POND water samples. All samples were placed in a light-tight container, stored on ice, and processed within 4 h of collection. Samples were filtered through 0.22-μm polycarbonate filters (Merck, USA). The volume filtered varied by sample type (INF, 20 mL; EFF, 100 mL; POND, 125 mL). Filters and SED aliquots (250 mg) were submerged in LifeGuard soil preservation solution (Qiagen, Germany) and stored at −80°C until DNA extraction.

### DNA extraction.

DNA was extracted using the PowerSoil Pro DNA extraction kit (Qiagen, Germany) according to the manufacturer's protocol. In brief, LifeGuard solution was removed by pipetting off from the filters, and the filters were subsequently bead beaten for 3 min at 3,000 oscillations per minute according to the manufacturer’s protocol (Mini-Beadbeater-24; BioSpec, USA). The supernatant was centrifuged (3,500 × *g*, 5 min), and the eluted DNA was stored at −80°C. A NanoDrop (ND-1000 spectrophotometer; Thermo Fisher Scientific, USA), Pico Green assay (Thermo Fisher Scientific, USA), and agarose gel electrophoresis were used to assess DNA quantity and quality. Three replicates per sample were processed with negative controls from the autosampler rinsates and controls of DNA extraction to test for contamination events.

### Sequencing.

The DNA was shipped frozen to Macrogen Oceania (South Korea). Library preparation was performed using a TruSeq Nano DNA library prep kit (Illumina, USA). Libraries were multiplexed and sequenced on the NovaSeq6000 platform (Illumina, USA), using 2 × 150-bp paired-end sequencing. The average data per sample generated were 11 Gb and 74 million reads. DNA extraction eluates of negative controls (rinsates of autosampler bottles and DNA extraction controls) were sequenced under the same conditions and did not contain significant amounts of DNA.

### Bioinformatics and statistics.

Sequence read quality control was performed with FastQC (v0.11.8) (78) and MultiQC (v1.11) (79), and then quality and length were trimmed with BBDuk2 (v38.90) (80). Kraken (v2.0.7) (81) and a database comprised of RefSeq complete genomes (including human) were used for taxonomic assignment, and assignments were further analyzed using phyloseq (v1.3.4) and vegan (v2.5-7) in R v4.0.5. Visualizations were created using the Interactive Tree of Life (iTOL) (82) and ggplot2 (v3.3.5). Alpha diversity normality was tested using Shapiro-Wilk tests, and significance was tested with nonparametric Wilcoxon signed-rank tests, with a *P* value of <0.05 deemed significant.

### Data availability.

The data generated in this study have been submitted to https://www.ncbi.nlm.nih.gov under BioProject accession number PRJNA904380.
